# Genome-Wide Characterization of the wnt Gene Family Reveals a *wnt5b*-Mediated Regulatory Mechanism of Testicular Development in *Cynoglossus semilaevis*

**DOI:** 10.3390/ani16030387

**Published:** 2026-01-26

**Authors:** Zhengjie Li, Junhao Wang, Chao Li, Ying Zhu

**Affiliations:** School of Marine Science and Engineering, Qingdao Agricultural University, Qingdao 266109, China; 15725362751@163.com (Z.L.); wjh3530200433xyl@163.com (J.W.); chaoli@qau.edu.cn (C.L.)

**Keywords:** WNT family, promoter, RNAi, wnt signaling pathway, sex differentiation

## Abstract

The wnt gene family encodes a group of highly conserved secreted glycoproteins that play essential roles in vertebrate development, including tissue patterning, cell differentiation, and gonadal regulation. However, the genomic organization, evolutionary dynamics, and functional roles of Wnt signaling components in flatfish remain poorly understood. In this study, we performed a comprehensive genome-wide identification, evolutionary characterization, expression profiling, and functional analysis of wnt genes in *Cynoglossus semilaevis*, a flatfish species exhibiting ZW/ZZ sex determination and temperature-induced sex reversal. A total of 20 wnt genes were identified and classified into 13 subfamilies, displaying conserved structural organization and phylogenetic relationships consistent with other teleosts. Chromosomal mapping revealed lineage-specific WNT clusters, including a unique wnt3–wnt7b–wnt5b–wnt16 block, as well as syntenic associations with reproduction-related genes (e.g., *adipor2*, *sema3a*, *nape-pld*, *erc2*, *lamb2*), suggesting coordinated genomic regulation. Tissue transcriptome analysis demonstrated strong sex- and tissue-biased expression patterns, with *wnt5a* predominantly expressed in ovaries and *wnt5b* specifically upregulated in pseudo-male testes. Functional assays revealed that knockdown of *wnt5a* or *wnt5b* induced testis-specific genes (*sox9b*, *tesk1*) and suppressed ovarian markers (*foxl2*, *cyp19a1a*), indicating antagonistic regulatory roles in gonadal fate determination. Promoter analysis identified *yy1a* as a selective repressor of *wnt5b*, but not *wnt5a*, providing a mechanistic basis for paralog divergence. Furthermore, pull-down combined with LC–MS/MS analysis showed that WNT5b interacts with proteins enriched in ribosome biogenesis and ubiquitin-mediated proteolysis, suggesting a role in translational regulation and protein turnover during spermatogenesis. Together, these findings establish WNT5 signaling—particularly *wnt5b*—as a key driver of testicular development in *C. semilaevis* and provide new insights into the molecular mechanisms underlying sex differentiation and sex reversal in flatfish.

## Simple Summary

We identified and analyzed 20 wnt genes in the Chinese tongue sole, a flatfish with ZW/ZZ sex determination and temperature-induced sex reversal. These genes showed conserved structures but also lineage-specific genomic features, including a unique wnt gene cluster linked to several reproduction-related genes. Expression analyses revealed strong sex-biased patterns: *wnt5a* is mainly expressed in ovaries, while *wnt5b* is highly expressed in pseudo-male testes. Functional experiments showed that reducing *wnt5a* or *wnt5b* pushes gonads toward a testis-like state, activating male genes and suppressing female markers. We further found that the transcription factor *yy1a* specifically represses *wnt5b*, explaining functional divergence between the two paralogs. Protein interaction analysis suggested that WNT5b is involved in ribosome biogenesis and protein turnover during spermatogenesis. Overall, our findings highlight wnt5—especially *wnt5b*—as a key regulator of testis development and sex differentiation in flatfish.

## 1. Introduction

WNT proteins constitute a highly conserved family of secreted signaling molecules that regulate fundamental developmental processes, including cell fate determination, proliferation, morphogenesis, and tissue patterning across metazoans [1,2]. Comparative genomic studies demonstrate substantial variation in wnt gene numbers across animal lineages. In mammals, 19 Wnt subfamilies with seven gene duplications have been identified, whereas only 7–9 members are present in arthropods such as *Apis mellifera*, *Drosophila melanogaster*, and *Tribolium castaneum*. In aquatic species, wnt gene complements also vary: *Danio rerio* possesses 19 Wnt genes [3], *Litopenaeus vannamei* carries 12 [4], and *Portunus trituberculatus* contains 13 [5]. Despite these differences, WNT proteins share a highly conserved Wnt domain and cysteine-rich motifs, underscoring their deep evolutionary stability and essential developmental functions [2,6].

WNT ligands activate multiple signaling pathways through interactions with conserved Frizzled (Fz) receptors and co-receptors on the cell membrane. In vertebrates, wnt genes are classified into thirteen subfamilies, which fall into three major signaling categories distinguished by the downstream cascades they trigger [7]. The canonical WNT/β-catenin pathway inhibits GSK3 activity, leading to the stabilization and nuclear translocation of β-catenin and subsequent regulation of genes involved in cell proliferation, differentiation, and gonadal development. The planar cell polarity (PCP) pathway signals through Rho and JNK/SAPK to govern cytoskeletal organization, cell polarity, and convergent extension movements. The WNT/Ca^2+^ pathway, another non-canonical branch, regulates intracellular calcium dynamics and influences processes such as cell migration and early germ-cell behavior [8,9]. Although originally characterized for their involvement in embryonic axis formation, WNT signaling has since been implicated in gonadal development, germ-cell specification, steroidogenic regulation, and natural or hormone-induced sex reversal across vertebrates. Increasing evidence indicates that individual WNT ligands exhibit pathway-specific functions relevant to sex differentiation in a taxon-dependent manner. For example, Wnt1, Wnt3a, and Wnt8 regulate axis specification in mice and Xenopus embryos [10,11]; Wnt2 is required for the survival of a male-specific subset of somatic gonadal precursor cells in the posterior gonad, acting synergistically with the JAK–STAT pathway to establish Drosophila melanogaster male gonadal identity [12]; and Wnt4 is essential for ovarian development and sex maintenance in vertebrates, including during sex change in black porgy (*Acanthopagrus schlegelii*) [13]. In vertebrates, WNT4 is a key determinant of ovarian formation and maintenance, whereas canonical ligands such as WNT1, WNT3a, and WNT8 regulate embryonic patterning through β-catenin signaling [14,15]. Among WNT ligands, WNT5a and WNT5b—representatives of non-canonical signaling—have attracted particular attention in teleost reproductive biology. These ligands are involved in primordial germ-cell migration, germ-cell proliferation, meiotic initiation, and somatic–germ cell communication in several fishes [16]. WNT5a often shows ovary-biased expression and has been linked to ovarian maintenance and germ-cell positioning [17]. But in some teleosts, such as zebrafish, *wnt5a* promotes the proliferation of testis via enriched type A spermatogonia [18], while *wnt5b* disrupts primordial germ cell migration and impairs male sexual development, consistent with the broader requirement for WNT signaling in regulating adult spermatogenesis in mice [19]. In contrast, *wnt5b* plays a more consistent role in male pathways. Accumulating evidence suggests that the two paralogs underwent functional divergence following genome duplication, contributing to species-specific differences in sex-related signaling.

Despite these advances, the genomic composition, evolutionary history, and reproductive functions of the wnt gene family remain poorly understood in flatfish. The Chinese tongue sole (*Cynoglossus semilaevis*), a commercially important flatfish species with a ZW/ZZ sex determination system, pronounced sexual dimorphism, and a high incidence of temperature-induced female-to-pseudomale sex reversal, provides a unique model for investigating the molecular mechanisms underlying gonadal plasticity. Sex reversal in *C. semilaevis* poses significant challenges for aquaculture because males exhibit markedly slower growth than females, yet the upstream signaling pathways contributing to masculinization remain incompletely characterized. While the involvement of WNT ligands, particularly the divergent WNT5 paralogs, has not been systematically examined in this species.

To fill these knowledge gaps, we conducted a comprehensive genome-wide identification and evolutionary analysis of the wnt gene family in *C. semilaevis*, coupled with chromosomal synteny mapping, motif and domain characterization, and tissue-specific expression profiling across females, males, and pseudo-males. Given the suspected roles of *wnt5a* and *wnt5b* in gonadal development, we further explored their functional divergence using RNA interference, promoter activity assays, subcellular localization, and in vitro expression studies. Finally, we applied pull-down proteomics (LC–MS/MS) to uncover the downstream protein interaction network of *wnt5b*, providing new mechanistic insights into how non-canonical WNT signaling intersects during testicular differentiation. Together, these analyses establish a comprehensive framework for understanding wnt gene evolution and highlight the key regulatory contributions of *wnt5* signaling to sex differentiation and sex reversal in flatfish.

## 2. Methods

### 2.1. Identification and Annotation of wnt Gene Family Members in C. semilaevis

To characterize the wnt gene repertoire in *C. semilaevis*, WNT protein sequences from a broad panel of vertebrates—covering mammals, birds, amphibians, and teleosts—were downloaded from the NCBI genome repository (https://www.ncbi.nlm.nih.gov/genome, accessed on 18 January 2026). These sequences were used to build a custom local BLAST database 2.17.0. Domain architecture and family annotations for WNT proteins were obtained from the Pfam database (http://pfam.xfam.org/). Putative wnt genes in *C. semilaevis* were first screened by BLAST searches against the genome assembly and further validated using HMMER to detect sequences harboring conserved WNT-specific domains, applying a stringent E-value cutoff of ≤1 × 10^−5^. Any sequences lacking essential conserved domains were removed from downstream analyses. Results from BLAST- and HMMER-based searches were subsequently merged to curate a complete and high-confidence set of wnt family members in the *C. semilaevis* genome for further evolutionary and functional characterization.

### 2.2. Phylogenetic Tree Analysis

To investigate the evolutionary relationships and structural characteristics of the wnt gene family, protein sequences from over 20 representative vertebrate species, including *C*. *semilaevis*, *Hippoglossus hippoglossus*, *Toxotes jaculatrix*, *Mus musculus*, and *Homo sapiens*, were retrieved from the NCBI protein database. Multiple sequence alignments were performed, and a phylogenetic tree was subsequently constructed using MEGA 11 employing default parameters unless otherwise specified. The resulting Newick format file was exported and further refined in the Interactive Tree of Life (iTOL) platform to enhance the clarity of clade organization and taxonomic annotation.

### 2.3. Structural Domain and Conserved Motif Analyses of the wnt Genes

To investigate the structural organization and evolutionary conservation of the *C. semilaevis wnt* gene family, conserved protein domains and motif architectures were systematically analyzed. Domain compositions of WNT proteins were identified using the SMART database (http://smart.embl.de/), and only domains supported by reliable e-value thresholds were retained. Conserved motifs were further characterized using the MEME Suite (https://meme-suite.org), with the maximum number of motifs set to ten. The domain and motif information obtained from SMART and MEME was subsequently integrated and visualized using TBtools V1.098, enabling comparative assessment of domain architecture, motif arrangement, and potential functional divergence among WNT family members.

### 2.4. Chromosome Distribution and Synteny Analysis of wnt Genes

Chromosomal localization and conserved synteny of Wnt family members were analyzed in eight vertebrate species, using *C. semilaevis* as the reference. For each species (*C. semilaevis*, *D. rerio*, *Scophthalmus maximus*, *H. hippoglossus*, *Carassius auratus*, *Channa argus*, *H. sapiens*, and *M*. *musculus*), the genomic positions of wnt genes and their neighboring genes were retrieved from the NCBI database. The order and orientation of *wnt* genes and their flanking genes were then compared across species to assess conserved syntenic relationships. The resulting chromosomal maps and synteny diagrams were arranged and finalized using Adobe Photoshop software 2023 (Adobe Inc., San Jose, CA, USA).

### 2.5. Transcriptomic Heatmap Analysis and qPCR Validation

To examine the transcript distribution patterns of *C. semilaevis* candidate genes, RNA-seq raw reads were retrieved from public repositories (NCBI SRA/ENA) based on previously published studies [20]. Expression values were normalized as TPM, and heatmaps were generated using TBtools to visualize tissue-specific expression profiles. For experimental validation of transcript abundance across tissues, quantitative real-time PCR (qPCR) was performed using gene-specific primers. Amplification was conducted under the following conditions: initial denaturation at 95 °C for 30 s, followed by 40 cycles of 95 °C for 5 s and 60 °C for 30 s. Melting curve analysis was applied to confirm primer specificity. Relative mRNA expression levels were calculated using the 2^−ΔΔCt^ method, with *β-actin* (or your reference gene) serving as the internal control.

### 2.6. RNA Interference and qPCR Analysis

The open reading frames (ORFs) of *wnt5a* and *wnt5b* from *C. semilaevis* were amplified using gene-specific primers to obtain full-length coding sequences. Based on the ORF regions, three siRNA candidates for each gene were designed using an online siRNA design tool. Ovarian cells were isolated and seeded into 24-well plates at an appropriate density. Cells were cultured under standard conditions (28 °C, 5% CO_2_), and transfection was performed when cell confluency reached approximately 50%. siRNA duplexes (final concentration 50–100 nM) were transfected using Lipo8000™ Transfection Reagent (Beyotime Biotechnology, Shanghai, China) following the manufacturer’s instructions. After 48 h, cells were harvested, total RNA was extracted using TRIzol reagent, and RNA quality was assessed by NanoDrop spectrophotometry (Thermo Fisher Scientific lnc, Waltham, MA, USA). cDNA was synthesized using a reverse transcription kit, and quantitative real-time PCR (qPCR) was carried out with SYBR Green chemistry to determine the knockdown efficiency of *wnt5a* and *wnt5b*. Relative gene expression levels were calculated using the 2^−ΔΔCt^ method. All experiments were conducted in triplicate with at least three biological replicates.

### 2.7. Dual-Luciferase Reporter Assay of wnt5a and wnt5b Promoters

Promoter regions of *wnt5a* and *wnt5b* were amplified based on the *C. semilaevis* genome. The *wnt5a* promoter fragment encompassed 1760 bp upstream and 180 bp downstream of the start codon (ATG), while the *wnt5b* promoter included 1880 bp upstream of ATG. Both fragments were cloned into the PGL3-basic luciferase reporter vector to evaluate their transcriptional activity. Potential transcription factor binding sites for CCAAT/enhancer-binding protein alpha (*cebpα*), pituitary-specific positive transcription factor 1 (*pou1f1a*), yin–yang 1a (*yy1a*), *c-jun*, and *myog* were predicted using the PROMO database (updated TF_8.3; accessed on 23 August 2023). Mutated promoter constructs were generated where predicted binding motifs were disrupted.

HEK293T cells were transfected with wild-type or mutant promoter plasmids using Lipofectamine 8000 (Beyotime, Shanghai, China) and maintained under standard culture conditions. After 48 h, cells were lysed, and luciferase activities were quantified using the Dual-Luciferase Reporter Gene Assay Kit (Beyotime). Relative promoter activity was calculated as the Firefly/Renilla luminescence ratio. Both *wnt5a* and *wnt5b* promoters exhibited significantly higher transcriptional activity compared with the negative control (PGL3-basic).

### 2.8. Subcellular Localization Analysis

To determine the subcellular localization of *wnt5a*, *wnt5b*, and *yy1a*, the expression constructs pcDNA3.1-*wnt5a*, pcDNA3.1-*wnt5b*, pcDNA3.1-*yy1a*, and the empty pcDNA3.1 vector were transfected into HEK293T cells using the Xfect™ transfection reagent (Clontech, Takara Bio, Japan) according to the manufacturer’s protocol. At 24–48 h post-transfection, cells were washed with PBS and fixed in 4% paraformaldehyde. Following fixation, nuclei were stained with 1 μg/mL DAPI, and the cell membrane was labeled with 3,3′-dioctadecyloxacarbocyanine perchlorate (DiO). After staining, coverslips were mounted using an anti-fade mounting medium. Fluorescence signals were observed and imaged using a Leica fluorescence microscope (Leica, Wetzlar, Germany) to assess the intracellular distribution of the expressed proteins.

### 2.9. Western Blot Analysis

Western blotting was performed to examine the protein expression of WNT5b in gonadal tissues of male, female, and pseudo-male *C. semilaevis*. Fresh gonad samples were homogenized in RIPA lysis buffer supplemented with protease inhibitors, and lysates were incubated on ice for 30 min followed by centrifugation at 12,000× *g* for 15 min at 4 °C. The supernatant was collected, and protein concentrations were determined using a BCA Protein Quantification Kit (Beyotime, China). Equal amounts of protein (20–40 μg per lane) were separated by SDS–PAGE and transferred onto PVDF membranes. After blocking with 5% non-fat dry milk in TBST for 1 h at room temperature, membranes were incubated overnight at 4 °C with primary antibodies against Wnt5b (1:1000 dilution). After washing, membranes were incubated with HRP-conjugated secondary antibodies (1:5000) for 1 h at room temperature.

### 2.10. Pull-Down Assay and LC–MS/MS Analysis

To identify proteins interacting with WNT5b, a pull-down assay followed by LC–MS/MS analysis was performed. Recombinant WNT5b protein was expressed and purified using a His-tag affinity system. For pull-down experiments, purified His-WNT5b was immobilized onto Ni–NTA agarose beads (Qiagen, Venlo, The Netherlands) and incubated with gonadal protein extracts from *C. semilaevis*. The clarified lysates were incubated with WNT5b-bound beads at 4 °C for 4 h with gentle rotation. Beads incubated with His-tag alone were used as negative controls. Eluted proteins were separated briefly by SDS–PAGE, and entire gel lanes were subjected to in-gel trypsin digestion. The resulting peptides were analyzed using an LC–MS/MS system equipped with a nano-UPLC and a high-resolution mass spectrometer. Proteins specifically enriched in the WNT5b pull-down relative to the control pull-down were considered putative WNT5b-interacting partners.

## 3. Results

### 3.1. Identification, Characterization and Chromosomal Distribution of wnt Genes in C. semilaevis

**Figure 1:**
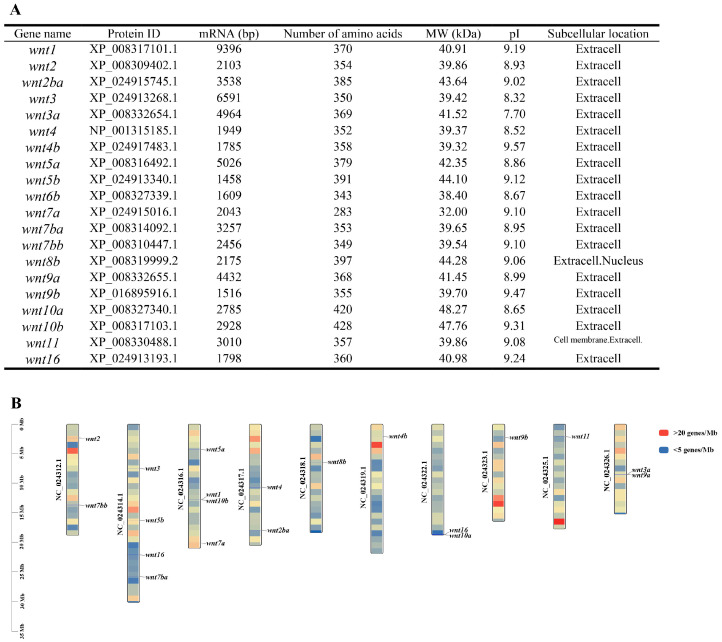
Characterization and chromosomal distribution of *wnt* genes in *C. semilaevis*. (**A**) Summary of the physicochemical properties of WNT proteins identified in *C. semilaevis*. For each gene, the corresponding protein ID, mRNA length (bp), number of amino acids, predicted molecular weight (MW, kDa), isoelectric point (pI), and subcellular localization are listed. (**B**) Chromosomal mapping of WNT genes in the *C. semilaevis* genome. Twenty *wnt* genes were distributed across nine chromosomes in a nonrandom pattern. Chromosome IDs are indicated on the left, and gene positions are shown according to their relative physical locations along each chromosome. Red indicates a gene density >20 genes/MB, while blue represents a gene density <5 genes/MB.

A total of 20 *wnt* genes were identified in the genome of *C. semilaevis* through homology-based searches and domain confirmation. These genes were designated as *wnt1*, *wnt2*, *wnt2ba*, *wnt3*, *wnt3a*, *wnt4*, *wnt4b*, *wnt5a*, *wnt5b*, *wnt6b*, *wnt7a*, *wnt7ba*, *wnt7bb*, *wnt8b*, *wnt9a*, *wnt9b*, *wnt10a*, *wnt10b*, *wnt11*, and *wnt16*. The full-length mRNA sequences of *wnt* genes ranged from 1458 bp (*wnt5b*) to 9396 bp (*wnt1*), encoding proteins of 283–428 amino acids. The predicted molecular weights (MW) varied between 32.00 kDa (WNT7a) and 47.76 kDa (WNT10b), while the theoretical isoelectric points (pI) ranged from 7.70 (WNT3a) to 9.57 (WNT4b). Subcellular localization prediction showed that the majority of WNT proteins were extracellular, consistent with their known role as secreted signaling molecules. Notably, *wnt8b* was predicted to localize in both the extracellular space and the nucleus, while Wnt11 was associated with both the cell membrane and extracellular compartments. The chromosomal mapping revealed that the *wnt* genes were unevenly distributed across nine chromosomes of *C. semilaevis* (Figure 1). Several *wnt* members were located in tandem or near each other, such as *wnt2* and *wnt2ba*, and *wnt7a* and *wnt7b*, suggesting potential gene duplication events during evolution. The *wnt1/2/3* genes were positioned in proximity, forming a conserved *wnt1/2/3* cluster similar to those reported in other teleosts.

### 3.2. Phylogenetic Analysis of the Wnt Family

**Figure 2:**
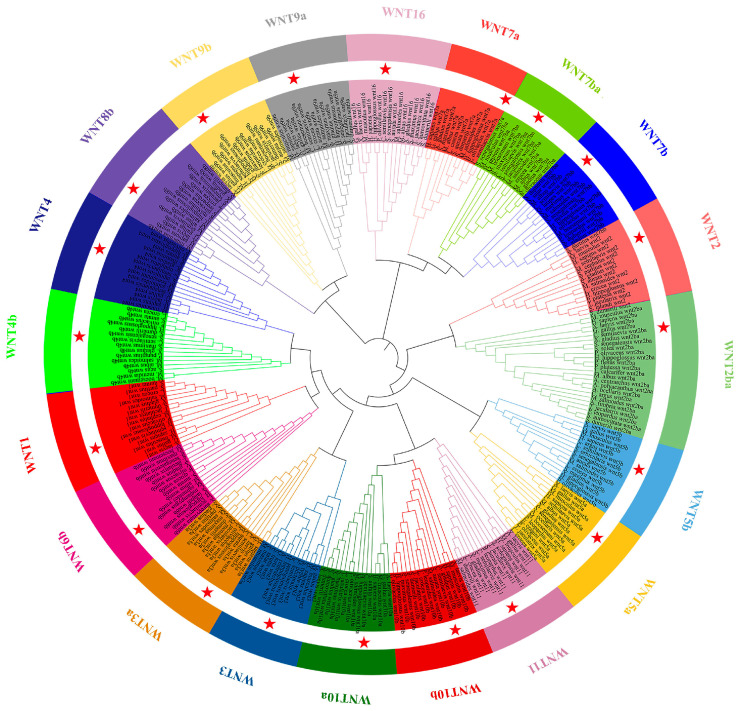
Phylogenetic relationships of wnt family genes among six teleosts and humans. A circular phylogenetic tree was constructed using the amino acid sequences of wnt family members from six teleost species (*C*. *semilaevis*, *M*. *salmoides*, *S*. *maximus*, *T*. *jaculatrix*, *H*. *hippoglossus*) and *H*. *sapiens*. The tree was generated using the maximum likelihood method with 1000 bootstrap replicates. Distinct colors represent different WNT subfamilies. Red stars mark *C. semilaevis* genes. Each subfamily forms a well-supported monophyletic clade, showing conserved evolutionary relationships among teleosts and vertebrates.

A phylogenetic tree was constructed based on the amino acid sequences of Wnt family members from six teleost species (*C. semilaevis*, *Micropterus salmoides*, *S*. *maximus*, *Toxotes jaculatrix*, *H*. *hippoglossus*) and *H*. *sapiens* to examine the evolutionary relationships among WNT proteins (Figure 2). The *wnt* genes were grouped into 13 well-defined subfamilies, including WNT1, WNT2, WNT2ba, WNT3, WNT4b, WNT5a, WNT5b, Wnt6, WNT7a, WNT7b, WNT9a, WNT9b, WNT10a/b, and Wnt11. Each subfamily was represented by orthologous members from different species, and the branching topology showed high bootstrap support within each clade. In *C. semilaevis*, all identified *wnt* genes were evenly distributed across the 13 subfamilies. The *wnt1*, *wnt3*, *wnt5a*, *wnt5b*, *wnt7a*, *wnt7b*, *wnt9a*, and *wnt11* subfamilies each contained a single *C. semilaevis* gene that clustered tightly with corresponding homologs from other flatfish species (*S. maximus* and *H. hippoglossus*). The *wnt2* and *wnt2ba* subfamilies were clearly separated, each containing distinct *C. semilaevis* sequences derived from the teleost-specific duplication of the ancestral *wnt2* gene. The WNTt4b clade was present in all teleosts examined but absent in *H*. *sapiens*, consistent with a teleost-specific retention event. Similarly, WNT10a and WNT10b formed two closely related yet independent clades, both containing *C. semilaevis* orthologs that grouped with their corresponding teleost counterparts.

### 3.3. Gene Structure and Conserved Motif Analysis of wnt Family Genes

**Figure 3:**
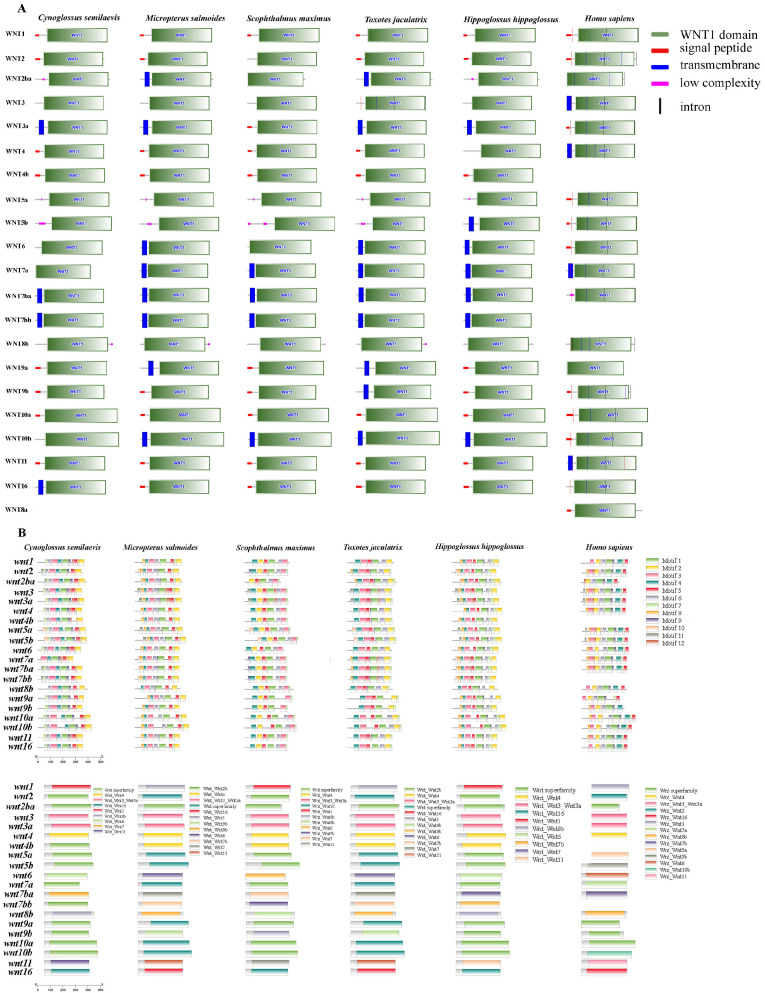
Gene structure and conserved motif analysis of wnt family genes in six species. (**A**) Exon–intron organization and conserved domain structures of Wnt genes in *C. semilaevis*, *Micropterus salmoides*, *S*. *maximus*, *T*. *jaculatrix*, *H*. *hippoglossus*, and *H*. *sapiens*. Exons (green boxes), introns (black lines), and untranslated regions (red boxes) show similar organization among orthologous genes, indicating high structural conservation. Conserved WNT domains are shown in blue, and transmembrane regions in pink. (**B**) Conserved motif patterns of WNT proteins identified using MEME. Each colored box represents a conserved motif, with identical colors corresponding to the same motif type.

To further characterize the structural organization and conserved features of the wnt gene family, exon–intron distribution and motif composition were analyzed across six species (*C. semilaevis*, *M*. *salmoides*, *S*. *maximus*, *T*. *jaculatrix*, *H*. *hippoglossus*, and *H*. *sapiens*). The exon-intron structures of wnt genes were generally conserved among all examined teleosts and humans (Figure 3A). The number of exons within *C. semilaevis* wnt genes ranged from three (*wnt4b*, *wnt5b*) to six (*wnt3a*, *wnt7b*), which is comparable to other teleosts and to *H*. *sapiens*. All *wnt* genes contained a single conserved WNT domain, which is characteristic of this family and essential for signal peptide secretion and receptor binding. Notably, *wnt10a* and *wnt10b* showed slightly longer coding regions due to extended introns, which may contribute to regulatory complexity and differential expression.

To elucidate the conserved sequence features of WNT proteins, motif composition was examined using the MEME suite (Figure 3B). A total of 10 conserved motifs were identified among all WNT proteins, and most paralogs shared highly similar motif arrangements. Across species, orthologous *wnt* genes displayed almost identical motif compositions, highlighting their evolutionary stability. For instance, WNT1, WNT3, WNT5a, and WNT7a exhibited complete conservation of all 10 motifs, while WNT8b and WNT9b displayed minor motif loss or truncation, possibly reflecting functional specialization. The motif pattern of *C. semilaevis* WNT proteins was nearly identical to that of *S. maximus* and *H. hippoglossus*, confirming the conserved architecture of WNT signaling components within flatfish.

### 3.4. Chromosomal Location of Wnt Family and Sex-Related Genes

**Figure 4:**
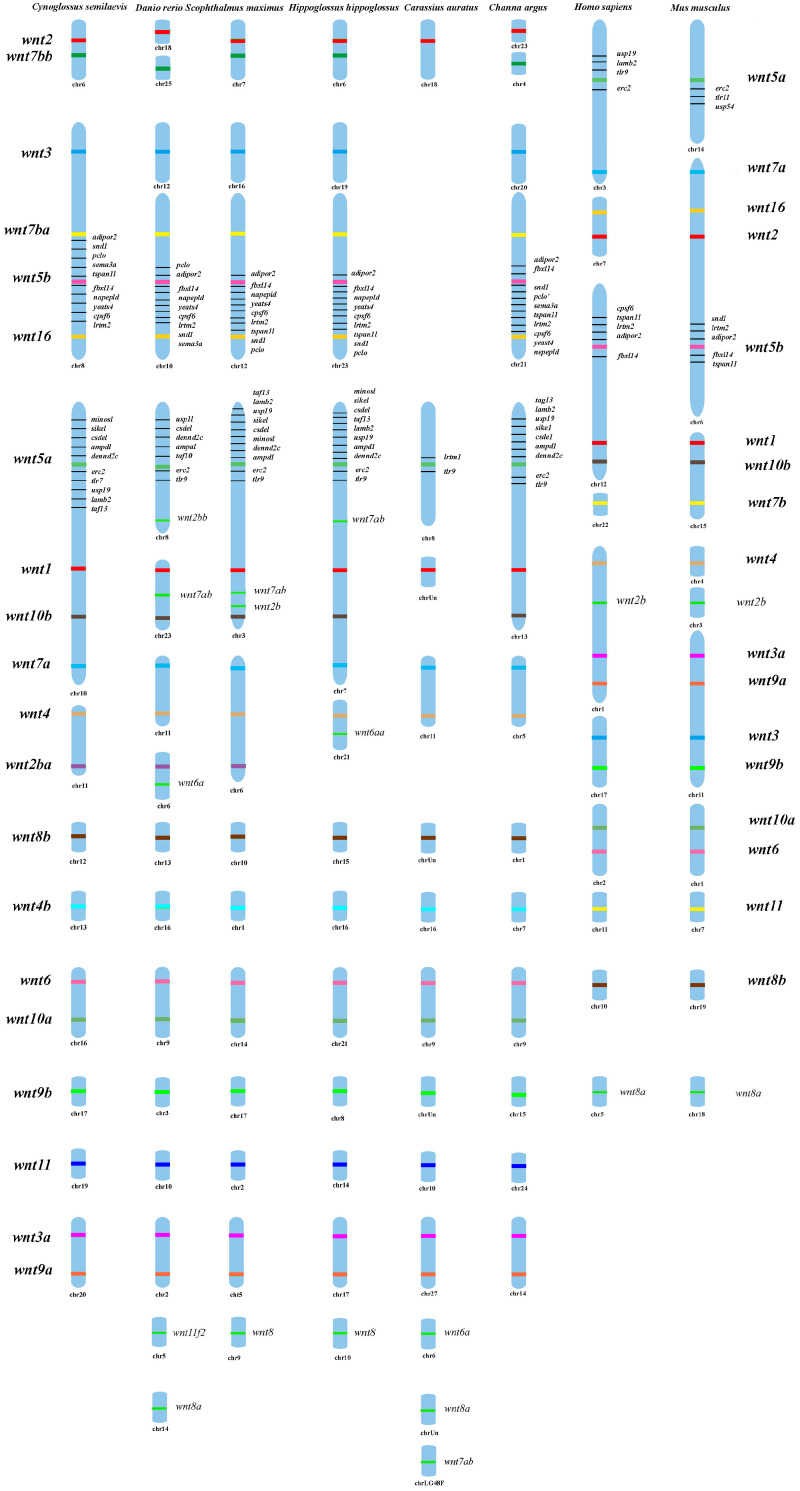
Chromosome distribution and synteny analysis of *wnt* genes among eight vertebrate species. Chromosomal localization of *wnt* genes in *C. semilaevis*, *D. rerio*, *S. maximus*, *H. hippoglossus*, *C. auratus*, *C. argus*, *H. sapiens*, and *M. musculus*. Colored bars represent chromosomes, and *wnt* gene positions are indicated along each chromosome.

To explore the chromosomal organization and evolutionary conservation of Wnt family genes, the genomic locations of *wnt* genes were compared across eight vertebrate species, including *C. semilaevis*, *D. rerio*, *S. maximus*, *H. hippoglossus*, *C. auratus*, *C. argus*, *H. sapiens*, and *M. musculus*. In *C. semilaevis*, *wnt* genes were distributed across multiple chromosomes, showing a species-specific localization pattern distinct from that of mammals. Several *wnt* genes exhibited conserved linkage relationships that were maintained in other teleosts but lost in higher vertebrates. Notably, *wnt2* and *wnt7b* were located on the same chromosome in most teleosts except *C. argus*. Interestingly, *wnt3*, *wnt5b*, *wnt7b*, and *wnt16* were found clustered on the same chromosome in *C. semilaevis*, while in other teleosts, *wnt3* was located independently, implying that genomic rearrangements may have occurred during flatfish genome evolution. Genes neighboring these Wnt loci, such as *erc2* and *lamb2*, have been implicated in gonadal differentiation and sexual development in teleosts. In most examined teleosts, *wnt4b*, *wnt8b*, *wnt3a*, and *wnt9b* were positioned on separate chromosomes, while their human and mouse orthologs showed more compact chromosomal clustering. Similarly, *wnt5a*, *wnt1*, *wnt10b*, and *wnt7a* were chained to the same chromosome in *C. semilaevis* and *H. hippoglossus*; particularly, they are clearly dispersed across different chromosomes in *D. rerio* (Figure 4).

### 3.5. Expression Profiles of Wnt Genes in Gonadal and Brain Tissues

**Figure 5:**
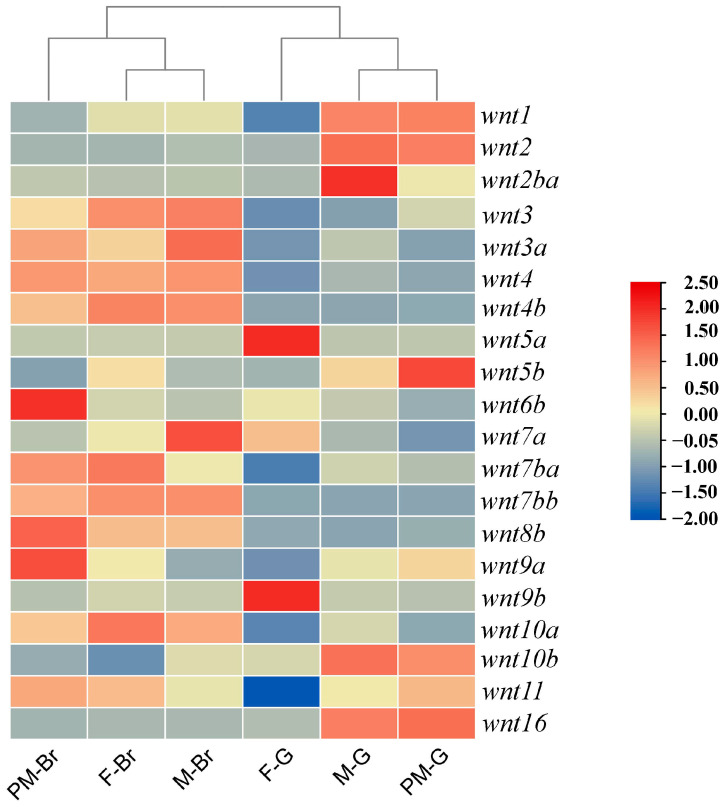
Expression profiles of wnt genes in gonadal and brain tissues of female, male, and pseudo-male *C. semilaevis*. Heatmap showing the relative expression levels of wnt family genes in brain (Br) and gonadal (G) tissues from females (F), males (M), and pseudo-males (PM). The color scale represents normalized expression (log_2_-transformed), with red indicating higher expression and blue indicating lower expression.

The expression patterns of wnt family genes were examined in the brain and gonadal tissues of female (F), male (M), and pseudo-male (PM) *C. semilaevis*. Overall, wnt genes exhibited tissue- and sex-specific expression differences, reflecting their potential functional diversification in neural and reproductive development. In the brain, several wnt genes, including *wnt1*, *wnt2*, *wnt3*, and *wnt9a*, showed relatively higher expression in brain tissues across all groups. In contrast, multiple wnt members displayed variable expression in gonadal tissues, with *wnt2b*, *wnt4b*, *wnt5a*, *wnt5b*, *wnt6*, and *wnt10a* displaying variable expression levels. Among them, *wnt5b* was markedly upregulated in the gonads of pseudo-males compared with both males and females, indicating a potential role in the masculinization process and testicular differentiation. Similarly, *wnt4b* also exhibited a moderate increase in male gonads, whereas *wnt10b* and *wnt7b* were more abundant in female gonads (Figure 5).

### 3.6. Expression Characteristics of wnt5a and wnt5b in Different Tissues of Male and Female C. semilaevis

**Figure 6:**
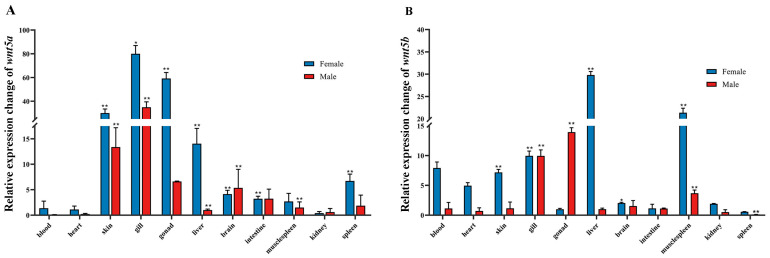
Tissue-specific expression profiles of *wnt5a* and *wnt5b* in male and female *C. semilaevis*. (**A**) The relative expression levels of *wnt5a* in various tissues (blood, heart, skin, gill, gonad, liver, brain, intestine, muscle, kidney, and spleen) of male and female *C. semilaevis*. (**B**) Relative expression levels of *wnt5b* in the same tissues of male and female *C. semilaevis*. Data are presented as mean ± SD (n = 3). Asterisks indicate statistically significant differences between sexes (one asterisk indicates *p* < 0.05, two asterisk indicates *p* < 0.01).

The tissue-specific expression profiles of *wnt5a* and *wnt5b* were analyzed in various tissues of adult male and female *C. semilaevis*, including blood, heart, skin, gill, gonad, liver, brain, intestine, muscle, kidney, and spleen. Overall, *wnt5a* transcripts were detected in all examined tissues, but expression levels varied significantly among organs. The highest expression was observed in the gill, followed by the gonad and skin, while only weak expression was detected in blood and heart. In females, *wnt5a* expression in the gill and gonad was significantly higher than in males (*p* < 0.01). *wnt5b* transcripts were detected in all examined tissues, with significantly higher expression levels in the gonad, liver, and muscle. The strongest expression was observed in the gonads of males. Compared with females, males exhibited markedly higher *wnt5b* expression in the gonad (*p* < 0.01) (Figure 6).

### 3.7. Functional Effects of wnt5a and wnt5b Knockdown on Sex-Related Gene Expression in C. semilaevis

**Figure 7:**
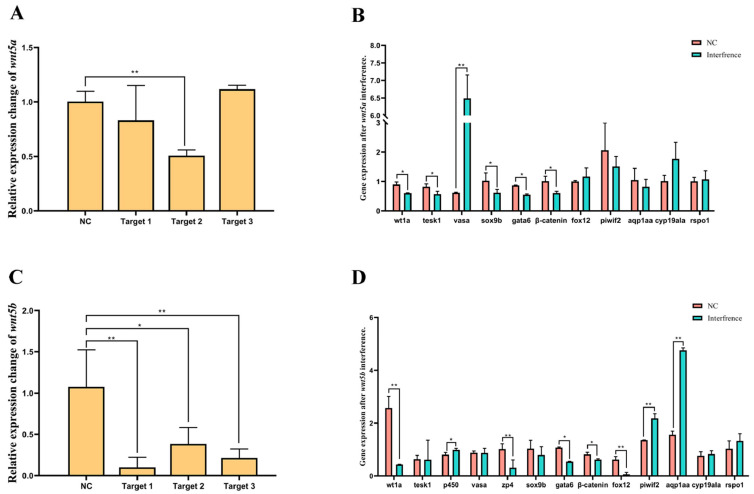
Effects of *wnt5a* and *wnt5b* knockdown on sex-related and germ cell–related gene expression in *C. semilaevis*. (**A**) Knockdown efficiency of three siRNA constructs targeting wnt5a. Target 2 showed the strongest suppression of wnt5a expression (*p* < 0.01). (**B**) Expression levels of key sex-related and germ cell–related genes after wnt5a interference. Testis-related genes (*wt1a*, *tesk1*, *sox9b*, *gata6*, *β-catenin*) and the germ cell marker (vasa) were significantly upregulated. (**C**) Knockdown efficiency of three siRNA constructs targeting *wnt5b*, with Target 1 exhibiting the highest interference efficiency (*p* < 0.01). (**D**) Gene expression changes following *wnt5b* knockdown. Similarly to *wnt5a*, testis-related genes (*sox9b*, *gata6*, *β-catenin*, *wt1a*) and germ cell markers (*vasa*, *zpl*) were significantly induced, whereas ovarian markers (*foxl2*, *cyp19a1a*) were suppressed. Data are presented as mean ± SD (n = 3). Asterisks denote significant differences relative to NC (one asterisk indicates *p* < 0.05, two asterisk indicates *p* < 0.01).

To elucidate the regulatory roles of *wnt5a* and *wnt5b* in gonadal differentiation, siRNA-mediated knockdown was performed, and the expression levels of key genes involved in sex determination, germ cell development, and gonadal maintenance were examined (Figure 7). To elucidate the regulatory roles of *wnt5a* and *wnt5b* in gonadal differentiation, siRNA-mediated knockdown was performed, and the expression levels of key genes involved in sex determination, germ cell development, and gonadal maintenance were examined. Similarly, *wnt5b* knockdown resulted in a strong reduction in transcript levels, with Target 1 showing the highest interference efficiency. Following *wnt5b* suppression, multiple testis-related genes—including *sox9b*, *gata6*, *dmrt1*, *β-catenin*, and *wt1a*—were also significantly upregulated, paralleling the transcriptional response observed after *wnt5a* knockdown.

### 3.8. Identification of Functional Promoters of wnt5a and wnt5b and Transcription Factor Regulation in C. semilaevis

**Figure 8:**
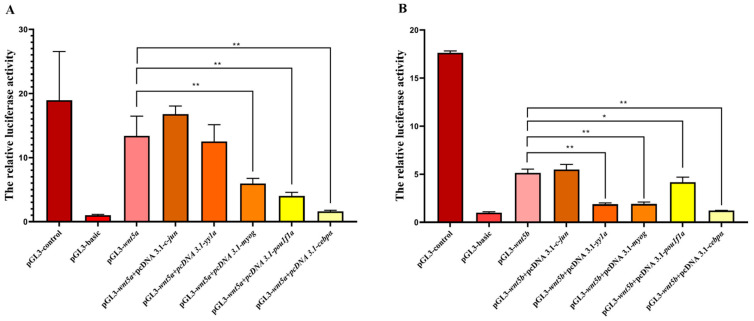
Transcription factor regulation of (**A**) *wnt5a* and (**B**) *wnt5b* promoters in *C. semilaevis*. Luciferase reporter assays depict the relative activity of pGL3-based constructs containing the upstream promoter regions of *wnt5a* and *wnt5b* under co-transfection with different transcription factors. Bars reflect mean ± SD (n ≥ 3). Asterisks denote significant difference compared with baseline promoter activity (one asterisk indicates *p* < 0.05, two asterisk indicates *p* < 0.01). Both promoters exhibited basal transcriptional activity above the pGL3-basic vector. Co-expression of *c-Jun* enhanced the activity of both *wnt5a* and *wnt5b*, while *myog*, *pou1f1a* and *cebpα* repressed both to varying degrees.

To characterize the upstream transcriptional regulation of *wnt5a* and *wnt5b*, their promoter regions were cloned into luciferase reporter vectors and evaluated for transcriptional activity. Both promoters showed significantly higher luciferase activity than the pGL3-basic vector, confirming that the cloned upstream sequences contain functional promoter elements. Although their basal activity was lower than that of the pGL3-control vector, the results clearly demonstrate that *wnt5a* and *wnt5b* possess intrinsic transcriptional activity. Co-transfection assays revealed that the two promoters respond differently to specific transcription factors. The transcription factor c-Jun strongly increased the activity of both *wnt5a* and *wnt5b*. Conversely, *myog* markedly suppressed the promoter activity of both genes, suggesting a conserved inhibitory role. Importantly, *yy1a* exhibited distinct regulatory effects on the two promoters. While *yy1a* significantly repressed the *wnt5b* promoter, resulting in a substantial decrease in luciferase activity, it showed no strong inhibitory effect on the *wnt5a* promoter, whose activity remained relatively stable in the presence of *yy1a*. This differential responsiveness indicates that *wnt5a* and *wnt5b* have undergone partial regulatory divergence, with *wnt5b* being more sensitive to yy1-mediated transcriptional repression. Other transcription factors, including *pou1f1a* and *cebpα*, displayed clear inhibitory effects on both promoters (Figure 8).

### 3.9. Subcellular Localization of Transcription Factor yy1a, wnt5a, and wnt5b

**Figure 9:**
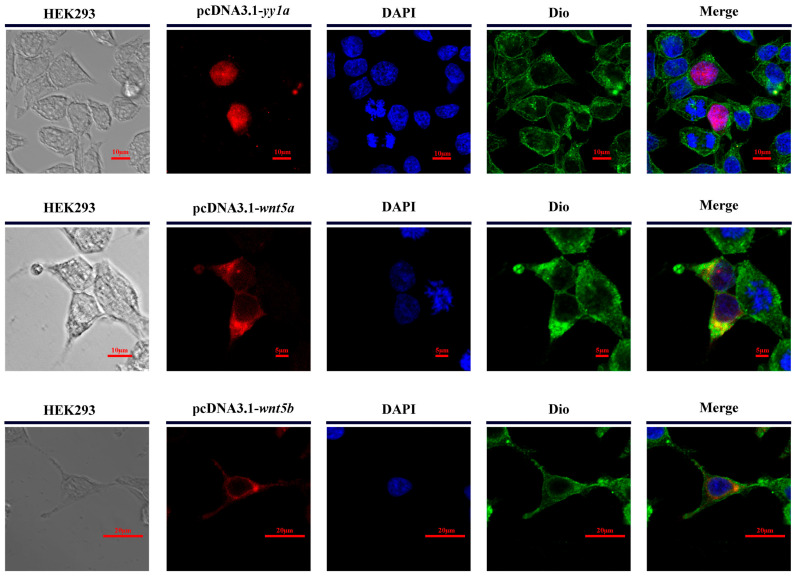
Subcellular localization of *Cs-yy1a*, *Cs-wnt5a*, and *Cs-wnt5b* in HEK293 cells. HEK293 cells were transiently transfected with pcDNA3.1-*yy1a*, pcDNA3.1-*wnt5a*, or pcDNA3.1-*wnt5b* constructs. Immunofluorescence detection shows yy1a (red) predominantly localized in the nucleus, with *wnt5b* at the nuclear envelope co-localizing with DAPI (blue). In contrast, *wnt5a* exhibits cytoplasmic and plasma membrane localization, partially overlapping with DiO-labeled membranes (green), indicated by yellow fluorescence in the merged images. Scale bars = 10 μm.

To determine the subcellular localization of Cs-yy1a, Cs-wnt5a, and Cs-wnt5b proteins, HEK293 cells were transiently transfected with pcDNA3.1 constructs expressing each gene. DAPI staining (blue) labeled the nucleus, while DiO staining (green) marked the plasma membrane and cytoplasmic regions. Distinct localization patterns were observed among the three proteins. The *Cs*-yy1a protein exhibited strong nuclear localization, with fluorescence signals almost completely overlapping with DAPI staining. In contrast, *Cs*-wnt5a displayed a clear cytoplasmic and membrane-associated localization, with fluorescence signals partially co-localizing with DiO staining. Notably, *Cs-wnt5b* showed a distinct localization pattern, accumulating primarily at the nuclear envelope rather than the cytoplasm or membrane and forming ring-like structures surrounding the nucleus (Figure 9).

### 3.10. Protein Expression and Detection of Recombinant WNT5b

**Figure 10:**
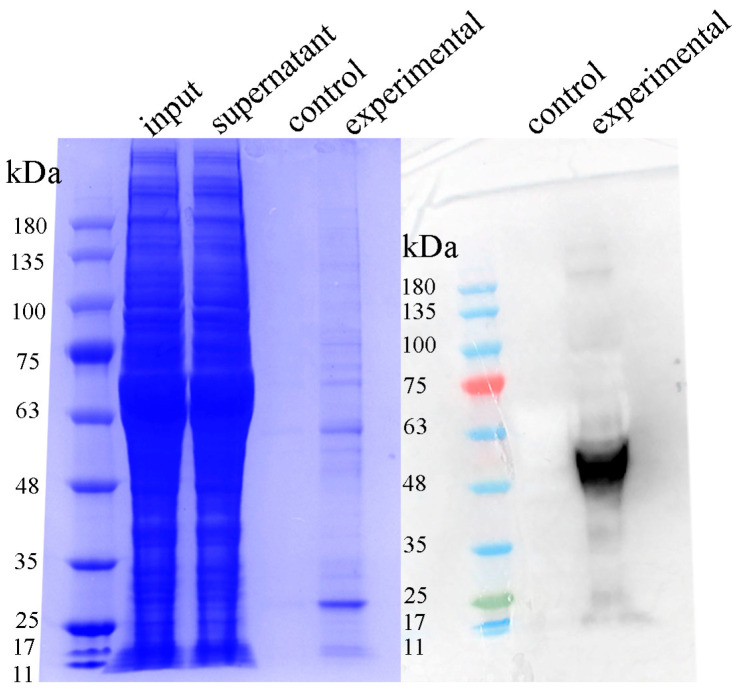
SDS–PAGE and Western blot analysis of recombinant WNT5b protein. Coomassie brilliant blue–stained SDS–PAGE showing a distinct protein band between 48 kDa and 63 kDa, corresponding to the expected molecular weight of recombinant WNT5b. (Right) Western blot analysis using the specific antibody detects a single immunoreactive band at the same position, confirming successful expression and detection of the WNT5b protein.

SDS–PAGE analysis revealed a clear protein band between 48 kDa and 63 kDa in the purified sample, corresponding to the expected molecular weight of the recombinant WNT5b protein. Coomassie blue staining showed a distinct enrichment of this band in the experimental lane, indicating successful expression and purification of the target protein. Consistently, Western blot detection using the specific antibody produced a strong, single immunoreactive band at the same molecular weight range, confirming the identity of the purified protein as WNT5b. No corresponding band was observed in the negative control lane. These results collectively verify the successful expression and accurate detection of recombinant WNT5b (Figure 10).

### 3.11. Functional Enrichment of WNT5b-Interacting Proteins

**Figure 11:**
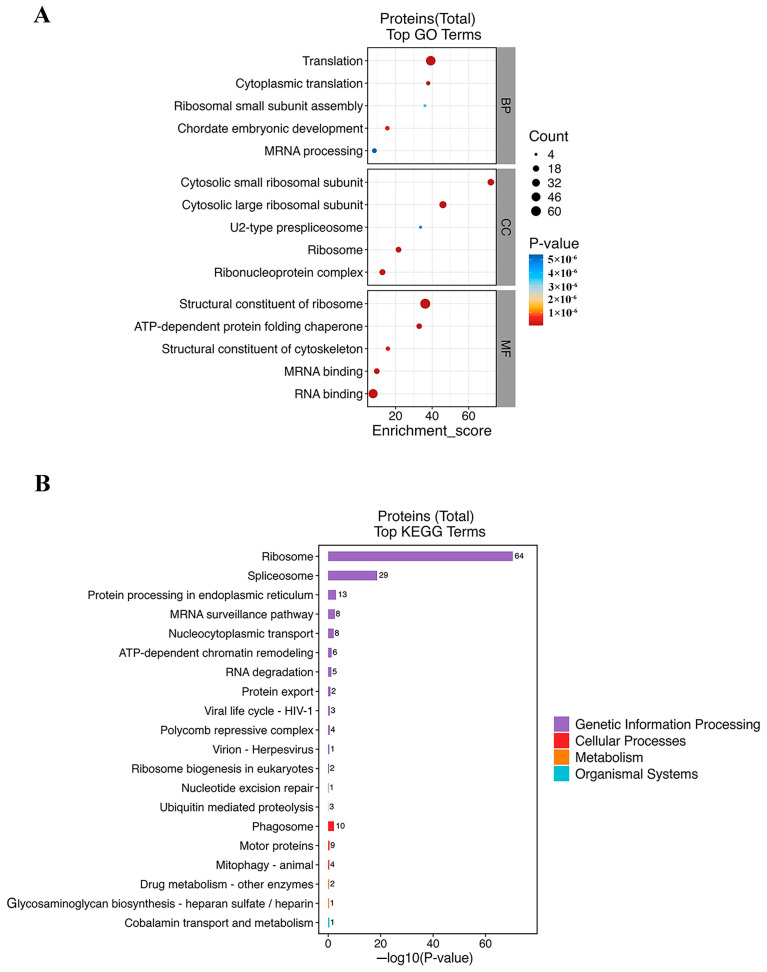
Functional enrichment of WNT5b-interacting proteins in *C. semilaevis*. (**A**) Gene Ontology (GO) enrichment analysis of proteins pulled down by WNT5b. The enriched terms span Biological Process (BP), Cellular Component (CC), and Molecular Function (MF) categories. Bubble size represents the number of enriched proteins, and color indicates the adjusted *p*-value. (**B**) KEGG pathway enrichment analysis of WNT5b-associated proteins. The most significantly enriched pathways include ribosome, spliceosome, protein processing in the endoplasmic reticulum, mRNA surveillance pathway, nucleocytoplasmic transport, and RNA degradation, reflecting strong involvement in genetic information processing. Additional enrichment was detected in ubiquitin-mediated proteolysis, ribosome biogenesis, mitophagy, motor protein function, and phagosome pathways. Bar length corresponds to enrichment significance (−log10 *p*-value).

To elucidate the molecular functions associated with WNT5b in *C. semilaevis*, the proteins pulled down by WNT5b were subjected to functional enrichment analysis using Gene Ontology (GO) and KEGG annotations. A total of WNT5b-associated proteins showed significant enrichment across multiple biological processes related to RNA metabolism, ribosome function, and protein synthesis. GO Biological Process analysis showed that several terms linked to early developmental events, such as chordate embryonic development, were also significantly enriched. Consistent with these findings. Consistent with these findings, the Cellular Component category indicated that many Wnt5b-associated proteins were localized to cytosolic ribosomal subunits, ribonucleoprotein complexes, and the U2-type prespliceosome. KEGG enrichment further supported that WNT5b-interacting proteins were highly concentrated in pathways associated with genetic information processing. Several cellular and metabolic pathways, including ubiquitin-mediated proteolysis, ribosome biogenesis, mitophagy, motor protein function, and phagosome formation, were also enriched (Figure 11).

## 4. Discussion

WNT proteins constitute a highly conserved family of secreted glycoproteins that act as key regulators of cell fate determination, proliferation, differentiation, and tissue patterning during embryonic development [21,22]. Comparative studies have demonstrated that wnt genes are broadly distributed across metazoans, including nematodes, insects, and vertebrates, and their amino acid sequences display remarkable evolutionary conservation, reflecting their indispensable role in developmental and morphogenetic processes [23]. Approximately 20 WNT family members have been reported in most vertebrate genomes, showing a highly conserved structure but diversified expression and functional regulation across species. In the present study, 20 Wnt genes (*wnt1*, *wnt2*, *wnt2ba*, wnt3, *wnt3a*, *wnt4*, *wnt4b*, *wnt5a*, *wnt5b*, *wnt6*, *wnt7a*, *wnt7ba*, *wnt7bb*, *wnt8b*, *wnt9a*, *wnt9b*, *wnt10a*, *wnt10b*, *wnt11*, *wnt16*) were identified in *C. semilaevis*. Comparative genomic evidence indicates that the number of wnt genes varies slightly among teleosts, but the overall gene complement remains stable. For instance, 11 Wnt genes have been identified in zebrafish (*wnt1*, wnt1-related (*zwntd*), *wnt2*, *wnt3*, *wnt4*, *wnt5b*, *wnt7b*, *wnt8*, *wnt8b*, *wnt10a* and *wnt11*) [24], while only five Wnt genes (*wnt4*, *wnt5a*, *wnt6*, *wnt7b* and *wnt8b*) have been characterized in medaka (*O. latipes*) [25]. Similarly, seven members (*wnt1*, *wnt3a*, *wnt4a*, *wnt5a*, *wnt5b*, *wnt8a*, and *wnt8b*) were detected in *Lutjanus malabaricus* [26]. Such variation in wnt gene copy number and subfamily composition likely reflects lineage-specific duplication or loss following the teleost-specific whole-genome duplication. Subcellular localization predictions indicated that most WNT proteins are secreted into the extracellular space, in agreement with their signaling function, while WNT8b and WNT11 were also predicted to localize to the nucleus or cell membrane, implying potential functional divergence in signal transduction and intracellular trafficking. The genomic distribution analysis revealed that wnt genes are unevenly dispersed across nine chromosomes, with several members (e.g., *wnt2*–*wnt2ba*, *wnt7a*–*wnt7b*) located in close proximity, suggesting segmental or tandem duplication events during teleost evolution. Notably, the *wnt1/2/3* cluster was well conserved, similar to other vertebrates, reinforcing the hypothesis that this ancient cluster has been evolutionarily preserved since the early diversification of the wnt family [27,28]. The conserved gene complement, coupled with species-specific subfamily expansions and chromosomal arrangements, underscores the evolutionary and developmental significance of WNT signaling in regulating key morphogenetic processes during flatfish development.

The phylogenetic relationships revealed a conserved composition and organization of the wnt gene family in *C. semilaevis*, consistent with the evolutionary patterns observed in other teleosts and vertebrates. A total of 20 WNT members were identified and classified into 13 subfamilies, suggesting that no large-scale gene loss or lineage-specific duplication occurred in *C. semilaevis* following the teleost-specific whole-genome duplication (3R). The close clustering of *C. semilaevis* wnt genes with their orthologs from Scophthalmus maximus and *H. hippoglossus* further highlights the strong evolutionary relatedness among flatfishes (*Pleuronectiformes*) and supports the notion that flatfish species have retained the ancestral WNT complement under strong purifying selection [29]. The wnt gene family has undergone extensive duplications and diversification during metazoan evolution; however, comparative genomic studies in zebrafish and medaka indicate that the overall repertoire remains relatively stable among teleosts, reflecting stringent evolutionary constraints on this development signaling pathway [27]. In vertebrates, 19 wnt genes have been described, of which at least five (WNT-1, WNT-2b, WNT-3a, WNT-8a, and WNT-8b) primarily signal through the canonical WNT/β-catenin pathway, two (WNT-4 and WNT-5a) function mainly through the WNT/Ca^2+^ pathway [30], and one (WNT-11) preferentially activates the WNT/JNK pathway, although cross-talk between pathways has been reported [31]. In *C. semilaevis*, the clear separation of *wnt10a* and *wnt10b* clades supports functional divergence between these paralogs, which may be associated with tissue-specific regulation in epithelial differentiation and skeletal development. Additionally, WNT5a, WNT7a, and WNT11 subfamilies showed high conservation among species, underscoring their fundamental roles in non-canonical WNT/PCP and WNT/Ca^2+^ signaling pathways that regulate tissue polarity, morphogenesis, and cellular homeostasis in vertebrates [32,33]. Collectively, these findings suggest that the wnt gene family in *C. semilaevis* exhibits high structural and evolutionary stability while preserving functionally diverse subfamilies that may contribute to morphological and developmental adaptations characteristic of flatfish. This conserved genomic framework may provide a molecular basis for understanding the regulatory mechanisms underlying asymmetric development and metamorphic remodeling in *C. semilaevis* and other flatfish species.

The chromosomal mapping analysis of the wnt gene family across seven vertebrate species revealed clear patterns of conserved synteny and lineage-specific rearrangements, highlighting both the evolutionary stability and genomic plasticity of WNT clusters in teleosts. In *C. semilaevis*, several wnt genes exhibited tight linkage on the same chromosome, such as the colocalization of *wnt2* and *wnt7b*, a pattern also retained in most examined teleosts except *C. argus* [34]. This configuration suggests that the ancestral teleost lineage likely maintained a conserved *wnt2*–*wnt7b* block that was later disrupted during species-specific chromosomal rearrangements in certain taxa. A particularly striking feature in *C. semilaevis* is the unique clustering of *wnt3*, *wnt7b*, *wnt5b*, and *wnt16* on the same chromosome. Such grouping was absent in other teleost species analyzed, where *wnt3* typically occupied an independent chromosome [28]. The teleost-specific whole-genome duplication (3R) event followed by extensive rediploidization has been shown to drive frequent chromosomal reshuffling, and the presence of this multi-gene WNT cluster in *C. semilaevis* may represent either a retained ancestral block or a derived recombination event that brought these genes into proximity. Similar WNT clustering phenomena have been reported in other vertebrates, such as the WNT3–WNT14B–WNT15 and WNT3A–WNT14 clusters in mammals [35,36]. Furthermore, several neighboring genes adjacent to wnt loci in *C. semilaevis*, including *erc2* and *lamb2*, have been implicated in gonadal development and sex differentiation in teleosts [37,38]. Similar genome architecture has been described in zebrafish, medaka, and tilapia, where clusters of sex-differentiation genes and WNT pathway components reside within syntenic blocks, suggesting conserved selective pressure toward co-regulation of developmental pathways. Another conserved pattern observed across teleosts involves the pairing of *wnt5a*, *wnt1*, *wnt10b*, and *wnt7a* on the same chromosome in both *C. semilaevis* and *H. hippoglossus*. Additional conserved synteny patterns were observed in the clustering of WNT4b, WNT8b, WNT3a, and WNT9b, paralleling the classical WNT3–WNT9B linkage observed in human and chicken genomes. These results support a model in which WNT clusters are evolutionarily constrained yet exhibit varying degrees of rearrangement across teleost lineages, consistent with previous comparative genomic studies [39,40].

The chromosomal localization analysis revealed that several genes positioned near the wnt gene family loci in *C. semilaevis* are functionally associated with sex differentiation, growth regulation, and gonadal development. These findings suggest that the genomic regions surrounding wnt genes may form functionally enriched neighborhoods that participate in reproductive and developmental regulation. For instance, *adipor2*, which was identified as a sex-specific gene in the hepatic protein–metabolite network of yellow catfish, was positioned near wnt-associated regions [41]. Similarly, *sema3a* was previously reported as a candidate growth-related gene in *Epinephelus coioides* and functionally linked to growth differences in *C. semilaevis*. The gene *nape-pld*, known to be expressed in Leydig cells and early meiotic cells in amphibians [42] and capable of regulating cell proliferation and apoptosis-related transcription [43], also mapped to the same chromosomal region. In addition, *erc2*, identified as a sex-associated genetic marker in *O. punctatus*, and *lamb2*, recognized as a key gene involved in sex differentiation and sex-determining signaling pathways, were positioned within syntenic blocks conserved across teleosts. Importantly, several genes adjacent to WNT loci are components or modulators of the WNT signaling pathway, which is well known for its essential roles in gonadal differentiation, somatic–germ cell communication, and tissue patterning, which suggests potential functional cooperation or coordinated regulation in sex differentiation.

The WNT signaling family plays evolutionarily conserved roles in vertebrate gonadal differentiation and brain-gonad axis regulation. In teleosts, WNT ligands exhibit strong sex-biased or tissue-specific expression patterns, functioning as regulatory cues during ovarian development, testis differentiation, and sex reversal processes [44,45]. In our study, the expression landscape of the 20 wnt genes in *C. semilaevis* revealed distinct transcriptional signatures across gonadal and brain tissues of females, males, and pseudo-males, reflecting both conserved and lineage-specific regulatory features. Consistent with previous findings in zebrafish, several canonical WNT ligands, such as WNT1, WNT3, WNT7a, and WNT9a, showed enriched expression in brain tissues regardless of phenotypic sex [45]. These patterns support the conserved role of WNT signaling in neurohypophysial hormones in the endocrine and paracrine control of gametogenesis in fish [46]. In the gonads, noncanonical WNT members displayed more pronounced sex-biased patterns. Notably, *wnt5a* and *wnt5b* showed divergent expression across sex phenotypes. *Wnt5a* exhibited female-biased expression, particularly in ovaries. In fact, loss of *wnt5a* disrupts primordial germ cell migration and male sexual development in mice [47]. In contrast, *wnt5b* exhibited a striking upregulation in pseudo-male gonads relative to both males and females. This finding diverges from previous reports in the Chinese soft-shelled turtle (*Pelodiscus sinensis*), where *wnt5b* shows higher expression in embryonic ovaries [48], suggesting that *wnt5b* may have undergone functional divergence in flatfish. According to previous reports, it shows that *wnt5b* plays roles in gonad development during early embryonic stages and is involved in testicular development in common carp (*Cyprinus carpio*) [49]. Collectively, our findings suggest that the wnt gene family retains a conserved backbone across teleosts, and the regulatory functions of specific paralogs, particularly *wnt5b*, may have undergone functional shifts in species, exhibiting an acquired masculinization-promoting role in flatfish.

The present study demonstrates that *wnt5a* and *wnt5b* function as key performers of testis differentiation programs in *C. semilaevis*. Interference of either gene resulted in highly consistent transcriptional shifts, characterized by the induction of testis-related genes (*sox9b*, *gata6*, *wt1a*, and *tesk1*) and the suppression of ovarian markers (*foxl2*, *cyp19a1a*, *P450aromA*). These findings support a model in which WNT5 signaling acts upstream of the canonical sex-determining cascade. In particular, *wnt5b* exhibited strong male-biased expression in pseudo-male testes, prompting us to examine its regulatory function through in vitro knockdown. The suppression of *wnt5b* led to significant upregulation of *tesk1* (Testis-specific protein kinase 1) and *aqp1aa* (AQP1 paralogous A), two genes essential for spermatogenesis. *Aqp1aa* is highly expressed in the germ cells of males and pseudo-males, with particularly strong expression in spermatids and spermatozoa [50]. Likewise, *tesk1*, which encodes testis-specific protein kinase 1, is expressed in spermatids and has been implicated in actin reorganization and cytoskeletal remodeling during spermiogenesis [51]. Phosphoproteomic analyses further identified Tesk1-like (*tesk1l*) as a potential regulator of β-catenin signaling in pseudo-males, suggesting that the Tesk–β-catenin axis may contribute to abnormal spermatogenesis and sex reversal in flatfish [52]. These observations are consistent with studies in other teleosts showing that *tesk1* participates in sex differentiation in tilapia [53] and is downregulated during steroid-induced gonadal feminization in scallops (*Patinopecten yessoensis*) [54]. In contrast, several ovarian-associated genes, including *foxl2*, *cyp19a1a*, and *P450aromA*, were significantly reduced following *wnt5b* interference. P450aromA (aromatase) is essential for estrogen synthesis and ovarian maintenance; its expression is typically high in ovaries and low in testes or hormonally masculinized gonads in teleosts. In black porgy (*Acanthopagrus schlegeli*), increased aromatase expression accompanies testicular degeneration and the onset of ovarian development during natural sex change [55]. Similar patterns are observed in the gobiid fish *Trimma okinawae*, where P450aromA is a key regulator during sex change [56]. The suppression of *cyp19a1a*/*P450aromA* after *wnt5b* knockdown therefore indicates a weakening of ovarian differentiation signals and a shift toward testis development. Integrating these findings, we propose that *wnt5b* functions as a regulatory switch that modulates the balance between male- and female-specific pathways. Its inhibition triggers a transcriptional environment conducive to spermatogenesis by (1) upregulating male pathway genes (*sox9b*, *tesk1*), (2) activating germ cell differentiation markers (*vasa*, *zpl*, *piwil2*), and (3) downregulating ovarian maintenance factors (*foxl2*, *cyp19a1a*). In summary, our study identifies *wnt5a* and *wnt5b* as critical regulators of sex differentiation in *C. semilaevis*. Their antagonistic control over male and female pathways underscores the evolutionary importance of WNT5 signaling across teleosts as a gatekeeper of gonadal fate. Further work exploring the downstream targets and signal integration (e.g., interplay with TGF-β, MAPK, or WNT/β–catenin pathways) will enhance our understanding of sex-reversal mechanisms in fish.

Promoter motif prediction revealed that *wnt5a* and *wnt5b* contain binding sites for several transcription factors, including *pou1fla*, *c-jun*, *myog*, *cebpα*, and *yy1a*, respectively. Among these, *yy1a* emerged as a major regulatory factor with markedly different effects on the two promoters. Dual-luciferase assays demonstrated that *Cs*-*yy1a* strongly repressed *wnt5b* promoter activity, whereas the *wnt5a* promoter exhibited no significant response to yy1a, indicating a clear functional divergence between the two *wnt5* paralogs. Such differential regulation aligns with their distinct expression profiles in *C. semilaevis*, where *wnt5b* is highly expressed in pseudo-male testes, while *wnt5a* predominates in ovaries, suggesting that *yy1a*-mediated repression of *wnt5b* may serve as a fine-tuning mechanism in male gonadal differentiation. In fact, YY1 has long been recognized as a multifunctional transcription factor essential for vertebrate gonadal development. In mammals, YY1 regulates steroidogenic gene expression and germ cell differentiation [57,58] and is required for proper spermatogonial maintenance in mice [59]. In chickens, YY1 controls testis development and mediates Sertoli cell function during spermatogenesis [57,58]. Evidence from teleosts further underscores YY1’s role in fish reproduction: yy1 in *C. semilaevis* indicated its involvement in gonadal development, potentially encompassing roles in gametogenesis [60]. Within this context, our finding—that YY1 selectively represses *wnt5b*, but not *wnt5a*—provides new insight into transcriptional partitioning between *wnt5* paralogs. *Wnt5b* is strongly associated with a potentially important role in gonad development of *Pelodiscus sinensis* during the early embryonic stages. Moreover, *wnt5b* promotes testis formation, and in common carp, *C. carpio*, non-canonical WNT signaling participates in germ cell differentiation [49]. Thus, YY1a-mediated modulation of *wnt5b* may represent a conserved regulatory switch promoting spermatogenic commitment while simultaneously suppressing ovarian pathways. Subcellular localization results further strengthen this regulatory model. *Cs*-*yy1a* was localized exclusively in the nucleus, consistent with its transcription factor function, whereas *Cs*-*wnt5a* was mainly cytoplasmic and membrane-associated, reflecting its secretory ligand nature. Notably, *Cs*-*wnt5b* displayed a unique perinuclear distribution enriched at the nuclear envelope.

Pull-down combined with LC-MS/MS analysis revealed that WNT5b interacts with a broad set of proteins enriched in pathways associated with ribosome biogenesis, protein processing, mRNA surveillance, and ubiquitin-mediated proteolysis. KEGG enrichment identified the ribosome pathway as the most significantly enriched category, consistent with the large number of ribosomal proteins (RPs) captured in the pull-down assay, including RPL5, RPL8, RPL9, RPL21, RPL23, RPS3, RPS6, RPS9, RPS12, and multiple additional members. Ribosomal proteins are indispensable components of ribosome assembly and protein synthesis and also exert important extraribosomal functions in cellular regulation [61]. A previous study reveals numerous ribosomal protein (RP) genes (including RPL5, 8, 9, 21, 23, and RPS 3, 6, 9, 12, et al.), which belong to the ribosome pathway, primarily regulate the co-translational folding of a specific group of male germ cell proteins that are crucial for sperm formation [62]. The strong enrichment of ribosome-related components in the WNT5b interactome suggests that WNT5b may influence testicular development through translational and post-transcriptional regulation rather than solely via canonical WNT signaling. Endocrine regulatory mechanisms also influence these pathways. For example, E2 treatment suppresses ribosomal activity during spermatogenesis in *Macrobrachium nipponense*, potentially contributing to sex reversal by altering ribosome biogenesis [62,63]. Additionally, some RPL members display female-biased expression in zebrafish gonads, further indicating that ribosomal subunits contribute to sexually dimorphic development [64]. Importantly, the WNT5b interactome was also enriched for ubiquitin-mediated proteolysis, a pathway essential for regulated protein turnover, signaling modulation, and removal of misfolded or damaged proteins. The ubiquitin–proteasome system (UPS) plays a well-established role in spermatogenesis, governing processes such as meiotic progression, chromatin remodeling, germ-cell apoptosis, and regulation of sex-related signaling molecules [65]. Together, the strong enrichment of ribosomal proteins and UPS components suggests that *wnt5b* may regulate male gonadal development in *C. semilaevis* by coordinating translational output and protein quality control. Given that *wnt5b* is highly expressed in pseudo-male testes, its interaction with ribosome- and ubiquitin-related proteins may contribute to pseudo-male development by modulating germ-cell protein production, turnover, and functional maturation.

In summary, our study provides a comprehensive characterization of the wnt gene family in *C. semilaevis* and reveals its pivotal involvement in flatfish gonadal differentiation and sex reversal. Comparative genomic and phylogenetic analyses demonstrate that the WNT repertoire of *C. semilaevis* is structurally conserved and organized into classical subfamilies, while exhibiting lineage-specific chromosomal clustering and syntenic associations with key reproduction- and growth-related genes (such as *adipor2*, *sema3a*, *nape-pld*, *erc2*, and *lamb2*). Tissue expression profiling further highlights pronounced sex- and tissue-biased transcriptional patterns, particularly for *wnt5a* and *wnt5b*, which show opposing expression trends in ovaries and pseudo-male testes, respectively. Functional assays indicate that WNT5 signaling acts as an important regulator of the gonadal fate: interference with *wnt5a*/*wnt5b* shifts the transcriptional landscape toward testis differentiation by upregulating male pathway genes and germ-cell markers while suppressing ovarian maintenance factors such as *foxl2* and *cyp19a1a*. Promoter analysis identifies *yy1a* as a differential upstream regulator that selectively represses *wnt5b*, providing a mechanistic basis for the transcriptional partitioning of *wnt5* paralogs. Finally, pull-down LC–MS/MS data reveal that WNT5b is embedded in interaction networks enriched for ribosome biogenesis and ubiquitin-mediated proteolysis, suggesting that it modulates spermatogenesis and pseudo-male development by coordinating translational output and protein quality control. Collectively, these findings establish *wnt5* as a key modulator of sex differentiation in *C. semilaevis* and propose a regulatory framework in which non-canonical Wnt signaling, transcription factor control, and ribosome/UPS pathways are integrated to govern flatfish gonadal development and sex reversal.

## 5. Conclusions

In conclusion, this study identifies WNT5 signaling as a key regulator of gonadal differentiation and sex reversal in *C. semilaevis*. The wnt family is evolutionarily conserved yet shows lineage-specific genomic organization. *wnt5a*/*wnt5b* exhibit contrasting sex-biased expression, and their disruption shifts transcription toward testis differentiation while suppressing ovarian maintenance genes. We further implicate *yy1a* in selective repression of *wnt5b* and link WNT5b to ribosome/UPS-related interaction networks. These results support an integrated model in which non-canonical WNT signaling coordinates transcriptional control and protein homeostasis during flatfish sex differentiation.

## Data Availability

The original contributions presented in this study are included in the article. Further inquiries can be directed to the corresponding author.
